# A prolonged activated partial thromboplastin time indicates poor short-term prognosis in patients with hepatic encephalopathy: insights from the MIMIC database

**DOI:** 10.3389/fmed.2025.1514327

**Published:** 2025-02-13

**Authors:** Liping Zhan, Yuping Yang, Biao Nie, Yanqi Kou, Shenshen Du, Yuan Tian, Yujie Huang, Ruyin Ye, Zhe Huang, Botao Luo, Lei Ge, Shicai Ye

**Affiliations:** ^1^Department of Gastroenterology, Affiliated Hospital of Guangdong Medical University, Guangdong Medical University, Zhanjiang, China; ^2^Department of Gastroenterology, The First Affiliated Hospital of Jinan University, Jinan University, Guangzhou, China; ^3^Department of Gastroenterology, Huanghe Sanmenxia Hospital, Sanmenxia, China; ^4^Department of Pathology, Guangdong Medical University, Zhanjiang, China; ^5^Department of Colorectal Surgery, Affiliated Hospital of Guangdong Medical University, Guangdong Medical University, Zhanjiang, China; ^6^Department of Gastrointestinal Surgery, Affiliated Hospital of Guangdong Medical University, Guangdong Medical University, Zhanjiang, China

**Keywords:** hepatic encephalopathy, activated partial thromboplastin time (APTT), prognosis, risk factors, database

## Abstract

**Objectives:**

This study investigates serum markers for short-term prognosis in hepatic encephalopathy patients.

**Background:**

Patients with hepatic encephalopathy face elevated mortality rates and bleak prognoses. However, effective prognostic models or indicators are lacking. This study aims to explore serum markers for predicting short-term prognosis in these patients.

**Methods:**

We conducted a retrospective analysis of 552 patients with hepatic encephalopathy, categorizing 429 individuals meeting exclusion criteria into normal and high activated partial thromboplastin time (APTT) groups. We assessed 12-day and 25-day survival rates using Kaplan–Meier analysis and Cox regression models to examine associations between groups and outcomes.

**Results:**

Upon comparing baseline characteristics, the high APTT group exhibited significant disparities in acute kidney injury, sepsis, coagulation disorders, and ascites (*p* < 0.05). In the multivariate COX regression model, the hazard ratios [HRs; 95% confidence interval (CI)] of 12- and 25-day mortality were 1.012 (1.001, 1.022, *p* = 0.033) and 1.010 (1.002, 1.018, *p* = 0.013), respectively. We discovered that APTT demonstrated an independent association with prognosis. Our findings revealed that the ability of APTT to predict short-term prognosis surpasses that of the traditional MELD model. Regarding 12- and 25-day survival, Kaplan–Meier survival curves from these groups demonstrated a lower survival probability for patients in the high APTT group than the normal group (log-rank *p* < 0.05). The results of subgroup analysis and interaction analysis indicate that APTT is not influenced by other confounding factors.

**Conclusion:**

A prolonged APTT suggests a poorer short-term prognosis in patients with hepatic encephalopathy.

## Introduction

Hepatic encephalopathy, a neurological disorder stemming from liver dysfunction, is characterized by cognitive impairment and altered consciousness, and its incidence has steadily risen in recent years (1–3). This condition presents a spectrum of neurological and psychiatric abnormalities, ranging from mild cognitive impairment to severe coma, often resulting in disorientation or profound debilitation for affected individuals (4, 5). Diagnosis of hepatic encephalopathy primarily hinges on clinical symptoms such as dizziness, confusion, lethargy, and coma, alongside the exclusion of alternative neurological disorders (6). The etiology of liver disease is a fundamental determinant of the progression and prognosis of hepatic encephalopathy, encompassing conditions such as cirrhosis and non-cirrhotic portal hypertension. Once diagnosed, prompt intervention is imperative. However, managing this condition not only imposes a financial burden on patients but also escalates societal expenses (7). Therefore, accurate assessment of prognosis is pivotal in guiding appropriate treatment strategies for these patients (8–10).

Despite ongoing research, the prognosis and management of hepatic encephalopathy remain inconclusive. Liver injury is known to diminish blood coagulation factors such as factors VII, IX, and X, leading to a finely balanced hemostatic state in affected patients (11). The liver plays a critical role in synthesizing blood coagulation factors, and both acute and chronic liver diseases, along with other factors, contribute to coagulation abnormalities. These abnormalities include reduced synthesis of coagulation and inhibitory factors, decreased clearance of activation factors, platelet deficiencies in both quantity and quality, hyperfibrinolysis, and increased intravascular coagulation. The propensity to bleed significantly heightens the risk of illness and mortality among patients with liver disease (12). While the international normalized ratio (INR) is strongly linked to the prognosis and severity of acute or chronic liver disease, it inadequately predicts bleeding risk and should not be relied upon solely for this purpose (13). The connection between hepatic encephalopathy and abnormalities in the coagulation system can be identified through routine and specialized thrombotests. Activated partial thromboplastin time (APTT), a widely used coagulation test, warrants further examination, particularly in patients with liver disease, as prolongation of APTT adversely impacts the prognosis of hepatic encephalopathy patients.

While hepatic encephalopathy is linked with liver dysfunction, portal hypertension, skeletal muscle issues, nutrition, and gut microbiome disturbances, unconventional assessments such as sarcopenia have demonstrated effectiveness in predicting outcomes (14–16). Despite the efficacy of these markers in prognosis prediction, relying solely on muscular steatosis for hepatic encephalopathy prognosis assessment proves impractical due to cost, patient compliance issues, and measurement complexities in clinical settings. If we can pinpoint a simpler-to-measure indicator with predictive performance akin to the MELD model, boasting high accuracy and resilience to confounding factors, early intervention in patient management could markedly enhance outcomes. Our aim was to identify a swift and precise indicator for assessing hepatic encephalopathy prognosis.

## Materials and methods

Utilizing database searches, we accessed data from the Medical Information Market for Intensive Care (MIMIC)-MEDI-IV database covering the period from 2012 to 2022. We focused on patients diagnosed with hepatic encephalopathy. The dataset retrieved from this database has undergone anonymization, ensuring that all personal patient information has been coded and any identifiable data excluded. Consequently, patient consent is deemed unnecessary for the utilization of this data (17, 18).

**Figure 1:**
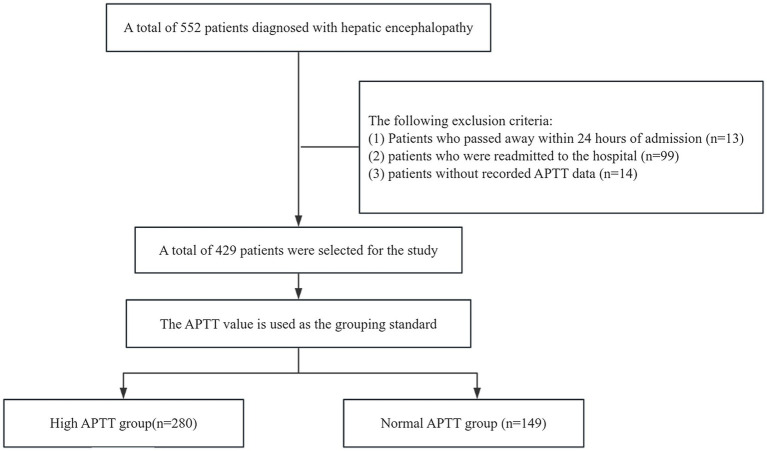
Flow diagram of group selection. (APTT: activated partial thromboplastin time).

In this retrospective study, we gathered data from the MIMIC IV database, comprising 552 patients diagnosed with hepatic encephalopathy. We applied the following exclusion criteria: (1) patients who deceased within 24 h of admission (*n* = 13); (2) patients with hospital readmissions (*n* = 99); (3) patients lacking recorded activated partial thromboplastin time data (*n* = 14). After applying these criteria, we identified a subset of 429 patients for analysis. The survival rates were observed at 12 days and 25 days. Based on their APTT values, which measure activated partial thromboplastin time, we categorized them into two groups. Given the normal APTT range of 23–36, individuals with a value exceeding 37 were included in the high APTT group, while those with APTT values falling within 23–36 were assigned to the normal APTT group. The high APTT group comprised 280 individuals, while the normal APTT group consisted of 149 individuals. For this study, we designated the high APTT group as the experimental group and the normal APTT group as the control group (Figure 1). Following consideration of potential confounding factors such as age, complications, and duration of hospital stay, we initially conducted a univariate analysis to identify risk factors for hepatic encephalopathy patients and potential confounders. Subsequently, we employed the COX regression model to comprehensively analyze all factors. We further strengthened our analysis by employing the Kaplan–Meier method for survival analysis, aimed at capturing survival rates among patients in the high APTT group compared to the normal group. Through comparison with MELD scoring systems via receiver operating curves (ROC), we assessed the predictive abilities of risk models for the 12- and 25-day survival of hepatic encephalopathy patients. The heatmap illustrates the distribution of activated partial thromboplastin time (APTT) across different levels of liver function impairment. Subgroup analysis revealed an interaction between APTT and various confounding factors in patients with hepatic encephalopathy.

### Data extraction

We collected data on various variables, including sex, age, length of hospital stay, length of ICU stay, routine biochemistry, presence of complications, and use of vasopressin. Subsequently, we compared scores of Logistic Organ Dysfunction System, Sequential Organ Failure Assessment, Autoimmune Polyglandular Syndrome Type III, Model for End-Stage Liver Disease, Oxford Acute Severity of Illness Score, Simplified Acute Physiology Score II, and Systemic Inflammatory Response Syndrome, between the experimental (high activated partial thromboplastin time) and control (normal activated partial thromboplastin time) groups. Our primary focus centered on studying the short-term survival time of patients within these groups. While the study cohort may include patients with both first-episode and recurrent hepatic encephalopathy, comprehensive pre-hospitalization data, including Child–Turcotte–Pugh and Model for End-Stage Liver Disease combined with serum sodium concentration scores, were not uniformly available.

### Data analysis

For comprehensive analysis and comparison between the two groups, we utilized SPSS version 27 (IBM SPSS) for statistical analysis. A bilateral *p*-value of less than 0.05 was deemed statistically significant. Numerical variables were described using mean for normally distributed data and median for non-normally distributed data, with normality assessed by the Shapiro–Wilk test. For normally distributed data, the *t*-test was used for group comparisons; for non-normally distributed data, the Mann–Whitney *U* test was applied. Qualitative data were summarized using percentages, with group comparisons conducted using the chi-square test or Fisher’s exact test where appropriate. Heatmaps were used to visualize data distribution and intergroup differences. Furthermore, Cox regression was employed to identify risk factors for hepatic encephalopathy, and the predictive power of the score was evaluated using receiver operating characteristic (ROC) curve analysis. To compare the AUC values between different prediction models, the DeLong test was employed. This test assesses the statistical significance of the AUC difference by estimating the standard error and covariance, thereby evaluating the significance of the performance differences between the models. Survival curves were compared using the log-rank test. Additionally, the test for comparing areas under ROC curves was performed to evaluate differences in predictive accuracy. Survival analysis was conducted utilizing Kaplan–Meier (KM) and Cox regression, with potential confounding factors taken into account.

## Results

### Participation in the baseline characteristics of patients

Initially, 552 patients were enrolled in the study. After applying specific exclusion criteria, a final cohort of 429 patients was included in the analysis. These patients were stratified into two groups based on their activated partial thromboplastin time (APTT) levels: the high APTT group (*n* = 280) and the normal APTT group (*n* = 149). The median APTT value for the normal group was 31.80 (IQR: 28.60–34.40) (95% CI: 30.19, 31.35), while the median for the high APTT group was 48.15 (IQR: 59.00–41.975) (95% CI: 53.07, 59.15). The two-sided *p*-value of the *t*-test was less than 0.001 at a significance level of 0.05, indicating a significant difference in APTT values between the two groups.

Regarding baseline characteristics, no statistically significant differences were observed between the high APTT and normal APTT groups in terms of sex, erythrocyte specific volume, mean erythrocyte hemoglobin concentration, potassium, Glasgow Coma Scale (GCS), and Oxford Acute Severity of Illness Score (OASIS) scores (*p* > 0.05) (Table 1). However, significant differences were noted between the two groups in terms of age and length of hospital stay, with patients in the high APTT group being younger and having longer hospitalization durations.

**Table 1:** Baseline characteristics of normal and high groups.

Factors	Normal APTT group	High APTT group	*p*-value
Age (year)	60.69 ± 12.18	55.91 ± 12.93	**<0.001**
Hospital stays	10.02 (14.99–5.83)	15.08 (26.27–1.73)	**<0.001**
INR	1.5 (1.7–1.3)	2.15 (2.9–1.8)	0.094
PT	16.70 (18.80–14.40)	23.10 (29.23–19.17)	**0.007**
GCS	15 (15–15)	15 (15–15)	0.281
LODS	15 (9–4)	7 (10–5)	**<0.001**
SOFA	7 (9–5)	9 (12–7)	**<0.001**
APSIII	63 (79–45)	74.5 (98.25–59.75)	**<0.001**
MELD	21.12 ± 7.58	31.04 ± 6.93	**<0.001**
OASIS	34 (71–29)	35 (43–25)	0.590
SAPSII	39 (48–33)	44 (55–34)	**<0.001**
SIRS	2 (3–2)	3 (3–2)	**<0.001**
Gender			
Man	102 (36.6%)	159 (59.3%)	0.408
Fenman	59 (63.4%)	109 (40.7%)	
Complications			
Diabetes			
Yes	49 (30.4%)	58 (21.6%)	0.042
No	112 (69.6%)	210 (21.6%)	
AKI			
Yes	84 (52.2%)	196 (73.1%)	**<0.001**
No	77 (47.8%)	72 (26.9%)	
Sepsis			
Yes	26 (16.1%)	81 (30.2%)	**0.001**
No	135 (83.9%)	187 (69.8%)	
Splenomegaly			
Yes	2 (1.2%)	3 (1.1%)	1.000
No	159 (98.8%)	265 (98.9%)	
Dialysis			
Yes	2 (1.2%)	7 (2.6%)	0.541
No	159 (98.8%)	261 (97.4%)	
Hyperlipidaemia			
Yes	38 (23.6%)	38 (14.2%)	**0.013**
No	123 (76.4%)	230 (85.8%)	
Hypertension			
Yes	22 (13.7%)	38 (14.2%)	0.882
No	139 (86.3%)	230 (85.8%)	
CKD			
Yes	31 (19.3%)	51 (19.0%)	0.954
No	130 (80.7%)	217 (81.0%)	
Pleural fluid			
Yes	11 (6.8%)	25 (9.3%)	0.367
No	150 (93.2%)	243 (90.7%)	
Ascites			
Yes	67 (41.6%)	164 (61.2%)	**<0.001**
No	94 (58.4%)	104 (38.8%)	
Coagulation disorders			
Yes	40 (24.8%)	123 (45.9%)	**<0.001**
No	121 (75.2%)	145 (54.1%)	
Heart failure			
Yes	22 (13.7%)	32 (11.9%)	0.602
No	139 (86.3%)	236 (88.1%)	
Portal hypertension			
Yes	71 (44.1%)	124 (46.3)	0.662
No	90 (55.9%)	144 (53.7)	
Cirrhosis			
Yes	123 (76.4%)	215 (80.2%)	0.348
No	38 (23.6%)	53 (19.8%)	
Dobutamine			
Yes	1 (0.6%)	2 (0.7%)	1.000
No	160 (99.4%)	266 (99.3%)	
Dopamine			
Yes	2 (1.2%)	12 (4.5%)	0.068
No	159 (98.8%)	256 (95.5%)	
Adrenaline			
Yes	2 (1.2%)	4 (1.5%)	1.000
No	159 (98.8%)	264 (98.5%)	
Norepinephrine			
Yes	35 (21.7%)	93 (34.7%)	**0.004**
No	126 (78.3%)	175 (65.3%)	
Isoprenaline			
Yes	17 (10.6%)	56 (20.9%)	**0.006**
No	144 (89.4%)	212 (79.1%)	
Antidiuretic hormone			
Yes	10 (6.2%)	43 (16.0%)	**0.003**
No	151 (93.8%)	225 (84.0%)	
Vasopressors			
Yes	43 (26.7%)	108 (40.3%)	**0.004**
No	118 (73.3%)	160 (59.7%)	
Oxygenation			
Yes	124 (77.0%)	207 (77.2%)	0.958
No	37 (23.0%)	61 (22.8%)	
Laboratory tests			
Haematocrit (%)	30.26 ± 6.70	29.11 ± 7.04	0.062
Haemoglobin (g/dL)	10.11 ± 2.29	9.71 ± 2.37	0.041
Platelets (×10^9^/L)	117 (168–69)	105 (162.25–69.75)	0.071
WBC (×10^9^/L)	8.20 (12.30–5.50)	9.95 (14.13–6.20)	**0.001**
MCH (pg)	32.12 ± 3.76	32.92 ± 3.60	**0.027**
MCHC (g/L)	33.28 ± 1.58	33.30 ± 1.86	0.953
MCV (fL)	96 (102–90)	99 (105–92)	**0.041**
RBC (×10^9^/L)	3.17 ± 0.74	3.00 ± 0.82	**0.011**
RDW (%)	16.50 (18.60–15.20)	17.50 (19.80–15.80)	0.587
Anion gap (mmol/L)	15 (18–12)	17 (20–14)	**0.007**
Bicarbonate (mmol/L)	23 (26–19)	20.5 (24–17)	0.088
BUN (mmol/L)	28.00 (47.00–16.00)	34.00 (56.25–18.00)	0.806
Calcium (mg/dL)	8.15 (8.80–7.50)	8.50 (9.20–7.70)	0.063
Chloride (mmol/L)	103.84 ± 8.34	101.69 ± 8.53	**0.002**
Creatinine (mmol/L)	1.00 (1.80–0.70)	1.70 (3.00–0.975)	**<0.001**
Glucose (mmol/L)	125 (161–100)	116 (148.5–94)	**0.018**
Sodium (mmol/L)	137.50 ± 6.43	125.60 ± 52.81	**<0.001**
Potassium (mmol/L)	4.20 (4.80–3.60)	4.1 (4.10–3.50)	0.780

The complications listed in the table are those that occurred during the admission of the patients with hepatic encephalopathy. The bolded numbers indicate significance at the *p*-value <0.05 level. INR, International Normalized Ratio; GCS, Glasgow Coma Scale; LODS, Logistic Organ Dysfunction System; SOFA, Sequential Organ Failure Assessment; APSIII, Autoimmune Polyglandular Syndrome Type III; MELD, Model for End-Stage Liver Disease; OASIS, Oxford Acute Severity of Illness Score; SAPSII, Simplified Acute Physiology Score II; SIRS, Systemic Inflammatory Response Syndrome; AKI, acute kidney injury; CKD, chronic kidney diseases; WBC, white blood cell count; MCHC, mean corpuscular-hemoglobin concentration; RBC, red blood cell count; RDW, red cell distribution width; BUN, urea nitrogen.

Additionally, significant disparities were identified between the high APTT and normal APTT groups in hematocrit, hemoglobin, platelet count, white blood cell count, mean corpuscular volume, mean corpuscular hemoglobin concentration, red blood cell count (Table 1).

Furthermore, differences were observed in the use of norepinephrine, isoprenaline, antidiuretic hormone, and vasopressin, as well as the prevalence of diabetes, acute kidney injury, coagulation disorders, ascites, hyperlipidemia, sepsis, and other complications between the two groups. Notably, scores for Logistic Organ Dysfunction System (LODS), Sequential Organ Failure Assessment (SOFA), Acute Physiology and Chronic Health Evaluation III (APSIII), and Model for End-Stage Liver Disease (MELD) were significantly higher in the high APTT group compared to the normal APTT group. The study included 91 patients without cirrhosis, reflecting the broader clinical context in which hepatic encephalopathy can arise from non-cirrhotic conditions. Within the subgroup of cirrhotic patients, elevated APTT remained an independent predictor of short-term prognosis, highlighting the robustness and applicability of our findings.

### Elevated APTT indicates poor short-term prognosis in patients with hepatic encephalopathy

**Figure 2:**
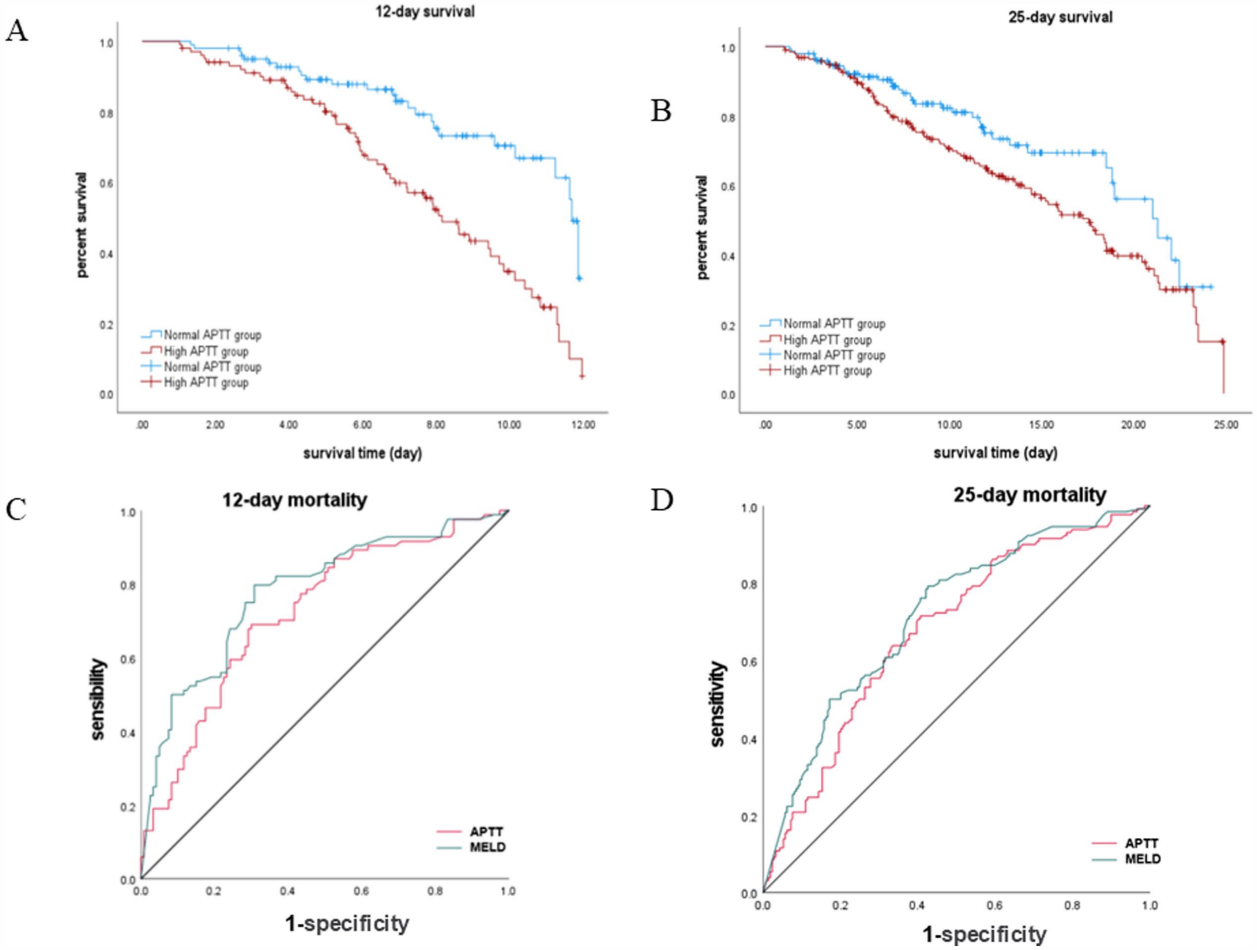
Survival analysis and ROC curve comparison for APTT and MELD in hepatic encephalopathy patients. **(A,B)** Kaplan–Meier survival curves stratified by APTT levels. **(C,D)** ROC curve analyses for predicting survival at 12-day and 25-day time points.

Our Survival plots figure revealed a significantly lower rate of survival for patients in the high APTT group, evident at both the 12-day and 25-day time points (Figures 2A,B). In the 12-day survival analysis, the log-rank (Mantel–Cox) *p*-value was found to be less than 0.001, indicating a robust statistical significance. Similarly, in the 25-day survival analysis, the log-rank (Mantel–Cox) *p*-value was 0.014, indicating a significant difference between the survival rates of the two groups.

### APTT predicts poor prognosis in patients with hepatic encephalopathy comparable to the traditional MELD model

Our analysis revealed that APTT exhibited comparable performance to the MELD score in predicting the survival of hepatic encephalopathy patients at both the 12-day and 25-day marks (Figures 2C,D). Specifically, for the 12-day mortality rate, the area under the curve (AUC) for APTT was 0.723 (95% CI: 0.652–0.794), while the AUC for MELD was 0.779 (*p* < 0.001). Similarly, for the 25-day mortality rate, the AUC for APTT was 0.679, and the AUC for MELD was 0.717 (95% CI: 0.621–0.736) (*p* < 0.001) (Table 2). For the ROC curve analyses, the area under the curve for the MELD model was higher than that for APTT at both evaluated time points. While the differences in AUC values were statistically significant (*p* < 0.05), the area under the curve for APTT remained close to that of the MELD model, suggesting that APTT provides a comparable level of predictive accuracy in assessing the survival of patients with hepatic encephalopathy.

**Table 2:** Receiver operating curves (ROC) for the abilities of risk models to predict 12- and 25-day survival of hepatic encephalopathy patients.

Factors	AUC	*p*-value	95% CI	
12-day mortality				
APTT	0.723	0.000	0.653	0.794
MELD	0.779	0.000	0.714	0.845
25-day mortality				
APTT	0.679	0.000	0.621	0.736
MELD	0.717	0.000	0.661	0.772

MELD, Model for End-Stage Liver Disease; APTT, activated partial thromboplastin time; AUC, area under the curve; CI, confidence intervals. ROC curve analysis for APTT and MELD in predicting survival in hepatic encephalopathy patients. The statistical comparisons of AUC values were conducted using the DeLong test.

### APTT emerges as an independent risk factor influencing the prognosis of patients with hepatic encephalopathy

In the unadjusted univariate model, significant differences were observed in APTT (*p* < 0.05) in both the 12-day and 25-day survival analyses, while no significant differences were noted in sepsis, hyperlipidemia, and coagulation disorders (*p* > 0.05). The hazard ratios [HRs; 95% confidence interval (CI)] for 12- and 25-day survival analyses were 1.011 (1.004, 1.017, *p* < 0.01) and 1.006 (1.000, 1.012, *p* = 0.039), respectively (Table 3).

**Table 3:** Multivariate Cox proportional regression model.

Factors	*p*-value	HR	95% CI	
Unadjusted				
APTT				
12-day survival analysis	**<0.01**	1.011	1.004	1.017
25-day survival analysis	**0.039**	1.006	1.000	1.012
Sepsis				
12-day survival analysis	0.935	0.978	0.575	1.664
25-day survival analysis	0.602	0.881	0.548	1.417
Hyperlipidemia				
12-day survival analysis	0.310	0.718	0.379	1.362
25-day survival analysis	0.803	0.928	0.518	1.664
Diabetes				
12-day survival analysis	0.142	1.440	0.885	2.341
25-day survival analysis	0.816	1.050	0.697	1.580
AKI				
12-day survival analysis	0.033	1.620	1.039	2.525
25-day survival analysis	0.877	0.969	0.654	1.438
Ascites				
12-day survival analysis	0.522	1.152	0.749	1.776
25-day survival analysis	0.267	1.222	0.858	1.739
Coagulation disorders				
12-day survival analysis	0.121	1.406	0.914	2.163
25-day survival analysis	0.210	1.249	0.882	1.769
Adjusted				
BUN				
12-day survival analysis	0.985	1.000	0.987	1.013
25-day survival analysis	0.104	1.007	0.999	1.016
Calcium				
12-day survival analysis	0.772	1.035	0.820	1.306
25-day survival analysis	0.267	0.899	0.745	1.085
Chloride				
12-day survival analysis	0.045	0.945	0.893	0.999
25-day survival analysis	0.349	0.982	0.946	1.020
Creatinine				
12-day survival analysis	0.498	1.071	0.879	1.305
25-day survival analysis	0.468	0.946	0.814	1.099
Sodium				
12-day survival analysis	0.083	1.063	0.992	1.139
25-day survival analysis	0.439	1.021	0.968	1.077
WBC				
12-day survival analysis	0.004	1.059	1.018	1.102
25-day survival analysis	0.231	1.014	0.991	1.037
MCH				
12-day survival analysis	0.236	1.798	0.682	4.743
25-day survival analysis	0.141	1.862	0.814	4.259
MCHC				
12-day survival analysis	0.229	0.559	0.217	1.441
25-day survival analysis	0.141	0.529	0.227	1.235
APTT				
12-day survival analysis	**0.033**	1.012	1.001	1.022
25-day survival analysis	**0.013**	1.010	1.002	1.018
Sepsis				
12-day survival analysis	0.408	0.772	0.418	1.425
25-day survival analysis	0.546	0.851	0.504	1.437
Hyperlipidemia				
12-day survival analysis	0.584	0.804	0.369	1.753
25-day survival analysis	0.764	1.108	0.567	2.168
Diabetes				
12-day survival analysis	0.071	1.696	0.955	3.010
25-day survival analysis	0.452	1.220	0.726	2.051
AKI				
12-day survival analysis	0.977	1.008	0.573	1.775
25-day survival analysis	0.376	0.804	0.497	1.302
Ascites				
12-day survival analysis	0.805	1.065	0.647	1.752
25-day survival analysis	0.711	1.088	0.697	1.697
Coagulation disorders				
12-day survival analysis	0.523	0.835	0.480	1.453
25-day survival analysis	0.160	1.383	0.879	2.176
Isoprenaline				
12-day survival analysis	0.093	1.736	0.913	3.303
25-day survival analysis	0.015	1.921	1.137	3.246
Gender				
12-day survival analysis	0.172	1.492	0.841	2.650
25-day survival analysis	0.475	1.176	0.754	1.834
Age				
12-day survival analysis	0.115	1.016	0.996	1.037
25-day survival analysis	0.285	1.010	0.992	1.029

CIs, confidence intervals; HRs, hazard ratios; BUN, urea nitrogen; WBC, white blood cell count; MCHC, mean corpuscular-hemoglobin concentration; AKI, acute kidney injury; APTT, activated partial thromboplastin time; MCH, mean corpuscular hemoglobin. Multivariate Cox proportional regression model was used to calculate HRs with 95% CIs. Model 1 was unadjusted. Model 2 was adjusted for age, gender, use of isoprenaline, patient’s past medical history of diabetes, sepsis, ascites, coagulation disorders, and other laboratory tests.The values marked in bold indicate that the *p*-values are less than 0.05, which means they are statistically significant.

Certain variables, such as sepsis, hyperlipidemia, diabetes mellitus, acute kidney injury, and ascites (*p* > 0.05), were intentionally excluded to mitigate collinearity (Table 3). Upon examination of indicators such as urea nitrogen, mean corpuscular hemoglobin concentration, and calcium ion concentration, APTT demonstrated an independent association with prognosis. The hazard ratios (HRs; 95% CI) for 12- and 25-day survival analyses were 1.012 (1.001, 1.022, *p* = 0.033) and 1.010 (1.002, 1.018, *p* = 0.013), respectively (Table 3).

### Analysis of APTT distribution across liver function impairment stages

**Figure 3:**
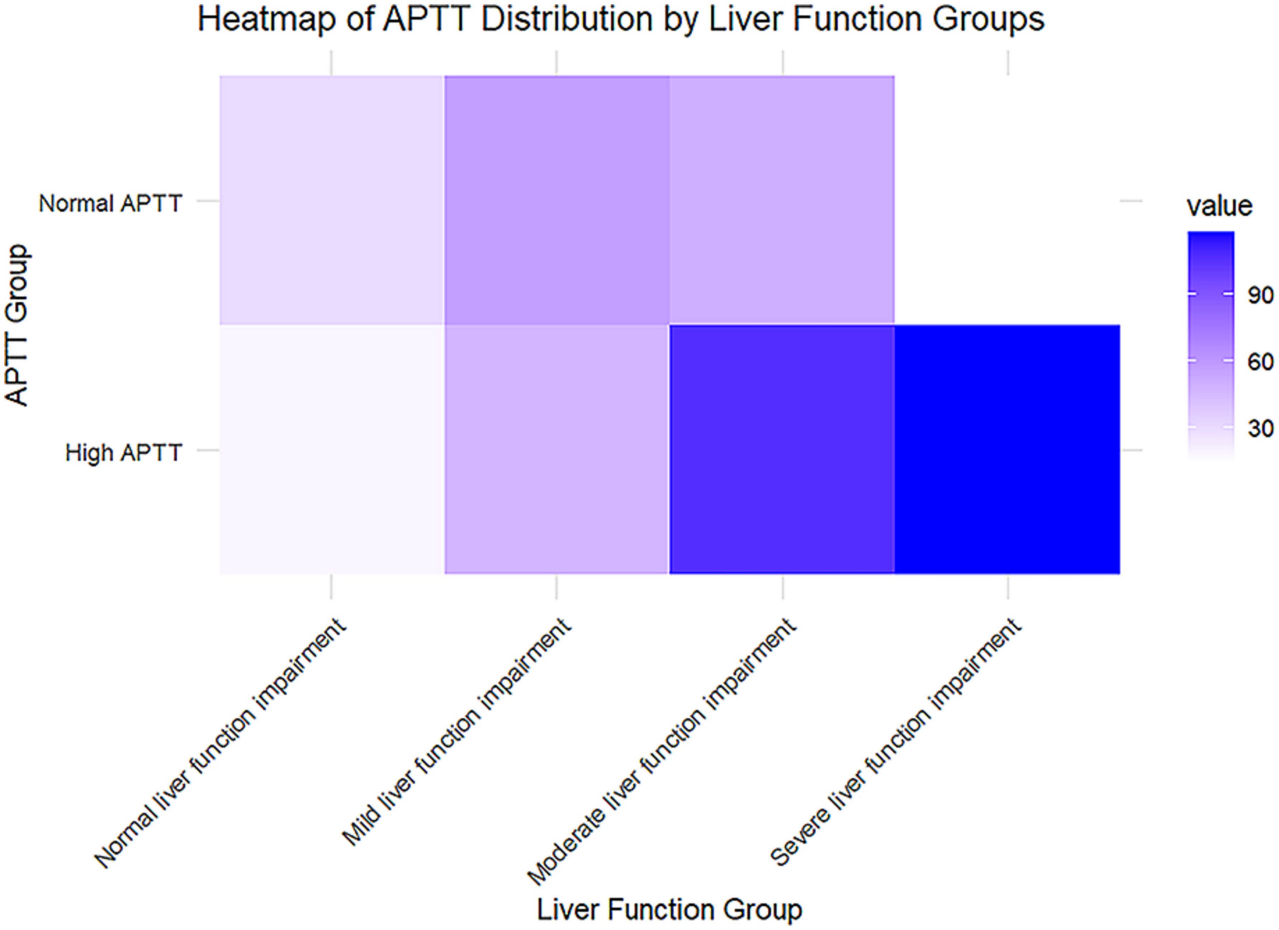
Heatmap of APTT distribution by liver function groups.

The liver function groups are categorized into four stages based on total bilirubin levels: normal liver function (≤1.2 mg/dL), mild impairment (1.2–3.0 mg/dL), moderate impairment (3.0–10.0 mg/dL), and severe impairment (>10.0 mg/dL). These classifications provide a clear framework for understanding the progression of liver dysfunction, with higher bilirubin levels indicating more significant liver impairment. By segmenting the data in this manner, the analysis aims to highlight potential variations in APTT across different stages of liver function deterioration, which can offer valuable insights into the coagulopathy associated with liver disease (Figure 3). The APTT is divided into normal and high categories, with color intensity representing the frequency or prevalence of cases in each subgroup. As liver function impairment progresses from normal to severe, there is a noticeable increase in the prevalence of high APTT, particularly in the severe liver impairment group, which is marked by the darkest color on the heatmap. This trend suggests a potential association between higher APTT values and worsening liver function.

### The results of subgroup analysis suggest that APTT predicts the short-term prognosis of patients with hepatic encephalopathy independently of other factors

Our analysis encompassed an examination of the interaction between APTT and various confounding factors in patients with hepatic encephalopathy. The findings reveal no significant interaction between APTT and confounding factors such as acute renal failure, hyperlipidemia, coagulation disorders, liver cirrhosis, and ascites (*p* < 0.05) (Table 4). This indicates that the APTT range remains unaffected by factors like liver cirrhosis or ascites, further underlining its role as an independent prognostic risk factor for patients with hepatic encephalopathy.

**Table 4:** Subgroup analysis of the associations between all-cause mortality.

Factors	*p*-value	HR (95% CI)	*p*-interaction
AKI			
No	1.000	1.000 (0.983, 1.018)	0.657
Yes	0.007	1.010 (1.003, 1.017)	
Hyperlipidaemia			
No	0.045	1.007 (1.000, 1.014)	0.064
Yes	0.035	1.022 (1.001, 1.042)	
Coagulation disorders			
No	0.269	1.005 (0.996, 1.013)	0.078
Yes	0.043	1.011 (1.000, 1.022)	
Cirrhosis			
No	0.314	1.007 (0.993, 1.021)	0.128
Yes	0.029	1.008 (1.001, 1.016)	
Ascites			
No	0.061	1.009 (1.000, 1.018)	0.979
Yes	0.148	1.006 (0.998, 1.015)	

CI, confidence intervals; HR, hazard ratios; AKI, acute kidney injury.

Differentiating acute-on-chronic liver failure, acute decompensation, and non-acute decompensation is crucial for understanding hepatic encephalopathy progression and prognosis. Due to data limitations, our study did not stratify patients by these categories. Future prospective studies should address this to better evaluate APTT and other prognostic markers.

## Discussion

Hepatic encephalopathy stands as the most prevalent complication in patients with advanced liver disease. While it’s commonly believed that the pathogenesis of hepatic encephalopathy is linked to elevated ammonia levels in the blood, the exact mechanisms remain inconclusive despite extensive literature (14, 19). Consequently, the use of blood ammonia as an indicator for predicting the prognosis of hepatic encephalopathy is subject to bias and lacks robust support from rigorous scientific literature. Patients with cirrhosis frequently exhibit abnormalities in hemostasis and coagulation indicators, including decreased platelet count, prolonged prothrombin time (PT), and reduced fibrinogen levels. Liver function abnormalities in these patients are often associated with a heightened risk of bleeding (11, 20). In normal individuals, there exists a delicate balance among the body’s coagulation system, anticoagulation system, and fibrinolytic system to prevent bleeding and thrombosis. However, patients with chronic liver disease or cirrhosis experience significant deviations from this balance (21). Firstly, abnormalities in platelet counts and function are prevalent in patients with chronic liver disease or cirrhosis. Reduced platelet counts, along with abnormal platelet function and decreased platelet aggregation ability, are common. Thrombocytopenia mechanisms include portal hypertension-induced platelet sequestration in the spleen, potential myelodysplasia suppression, immune-related platelet destruction, and diminished thrombopoietin production, primarily synthesized by the liver (22, 23). Secondly, liver disease disrupts the synthesis of various blood coagulation factors, as the liver is the primary site of synthesis for nearly all coagulation factors except Von Willebrand factor (VWF). Consequently, disorders in the synthesis of factors II, V, VII, IX, X, and XI occur (24, 25). Lastly, abnormalities in fibrinogen function and synthesis, along with hyperfibrinolysis, are common in cirrhosis patients. These alterations significantly increase the risk of bleeding, subsequently elevating the risk of disease progression and mortality in patients with hepatic encephalopathy (25–27).

Following the initial single-variable screening, multivariate analysis was employed to accurately assess the relationship between predictors and the dependent variable. Through this comprehensive analysis, it becomes evident that activated partial thromboplastin time (APTT) serves as a robust predictor of the prognosis of hepatic encephalopathy. Patients with elevated APTT values tend to experience longer hospital stays and higher mortality rates compared to those with normal APTT values. Moreover, older patients with higher APTT values are at an increased risk of developing hepatic encephalopathy and facing elevated mortality rates. The hazard ratios for APTT [1.011 (1.004–1.017) for 12-day survival and 1.006 (1.000–1.012) for 25-day survival] demonstrate a statistically significant association with short-term prognosis in hepatic encephalopathy patients. Although the effect sizes are modest, these findings suggest that even small elevations in APTT may reflect critical underlying coagulopathy or systemic dysfunction, which are known to contribute to worse outcomes in liver disease. The ROC curve analysis demonstrated that while the MELD model showed statistically higher AUC values compared to APTT, the differences were relatively small, indicating that APTT has a predictive performance comparable to MELD in assessing the survival of patients with hepatic encephalopathy. These results suggest that APTT could serve as an alternative or complementary predictor to the MELD model. Further investigations are warranted to explore potential synergies between APTT and MELD in improving prognostic accuracy. The prevalence of elevated APTT was significantly higher, particularly in the severe liver dysfunction group. This trend suggests a potential association between higher APTT values and liver function deterioration, indicating that APTT may serve as a useful prognostic marker for liver disease, especially in conditions like hepatic encephalopathy. From a clinical perspective, APTT serves as a readily available and cost-effective test that can be incorporated into routine practice to stratify risk and guide early interventions. However, its modest effect size indicates that APTT should be considered as part of a multifactorial prognostic assessment rather than a standalone marker. Additionally, the high APTT group exhibits a greater propensity for complications such as acute kidney injury, sepsis, coagulation disorders, and ascites. These patients often require more intensive medical interventions, including the frequent use of medications such as norepinephrine, isoprenaline, antidiuretic hormone, vasopressin, and ventilators. Such complexities in their condition contribute to prolonged treatment durations and extended hospital stays. In identifying independent risk factors for the prognosis of hepatic encephalopathy while considering various confounding factors, the COX regression model highlighted APTT as a significant independent risk factor. Complications such as acute kidney injury, sepsis, coagulopathy, and ascites were found not to be independent risk factors, indicating no linear correlation between these complications and the prognosis of hepatic encephalopathy. Furthermore, APTT demonstrates comparable predictive efficacy to the laboratory-based Model for End-Stage Liver Disease (MELD) model. Kaplan–Meier survival analysis further substantiates the prognostic significance of APTT, revealing a marked decrease in survival among patients with elevated APTT values. Importantly, the simplicity, convenience, and cost-effectiveness of APTT measurement make it an attractive option for clinicians. This not only conserves medical resources but also enhances patient compliance and improves treatment outcomes. In conclusion, APTT emerges as a valuable and accessible tool for forecasting the prognosis of patients with hepatic encephalopathy, contributing to more effective clinical management and better patient outcomes.

Moreover, APTT showcases comparable predictive efficacy to the laboratory-based Model for End-Stage Liver Disease (MELD) model. Kaplan–Meier survival analysis further reinforces the prognostic significance of APTT, indicating a significant decrease in survival rates among patients with elevated APTT values. Significantly, the simplicity, convenience, and cost-effectiveness of APTT measurement render it an appealing option for clinicians. This not only conserves medical resources but also enhances patient compliance and facilitates improved treatment outcomes. But the dynamic and complex nature of disease decompensation, as highlighted by the CANONIC and PREDICT studies, underscores the importance of understanding baseline patient status before hospitalization. In our cohort, baseline data such as pre-hospital liver function, previous decompensation episodes, and outpatient management history were not uniformly recorded, limiting our ability to fully assess the influence of pre-hospital factors on APTT levels and outcomes. Future studies should aim to incorporate comprehensive baseline data to provide a more holistic understanding of the progression and prognostic markers of hepatic encephalopathy. Anyway, APTT emerges as a valuable and accessible tool for predicting the prognosis of patients with hepatic encephalopathy, thereby contributing to more effective clinical management and better patient outcomes.

## Conclusion

A prolonged APTT suggests a poorer short-term prognosis in patients with hepatic encephalopathy.

## Data Availability

The raw data supporting the conclusions of this article will be made available by the authors, without undue reservation.
